# Perception of and risk factors for type 2 diabetes among students attending an upstate New York college: a pilot study

**DOI:** 10.1186/s13098-020-00535-1

**Published:** 2020-03-30

**Authors:** Janet Antwi, Rebecca Lavin, Stacey Sullivan, Maria Bellavia

**Affiliations:** 1 Department of Agriculture, Nutrition and Human Ecology, Nutrition and Dietetics, Prairie View A&M University, 100 University Dr, Prairie View, TX 77446 USA; 2 Department of Human Ecology, Nutrition and Dietetics, State University of New York at Oneonta, Oneonta, NY USA

**Keywords:** Type 2 diabetes, Perception, Risk factors, College students, Upstate New York

## Abstract

**Background:**

Detecting early type 2 diabetes (T2D) risk factors may reduce or prevent the development of the disease. We conducted a pilot study to generate preliminary data on the perception of T2D and further determined the prevalence of T2D risk factors among college students at an upstate New York campus.

**Methods:**

Metabolic profiles were available for 44 college students for cross-sectional analysis. The American Diabetes Association screening guidelines were used to determine risk factors, and perceived susceptibility, perceived seriousness, and self-efficacy were determined with the Health Belief Model’s constructs. Sociodemographic and anthropometric data, nutrition knowledge, and metabolic profiles were obtained.

**Results:**

The most common T2D risk factors were lack of physical activity (61.4%), decreased high-density lipoprotein cholesterol (HDL-c, 56.8%), high fasting blood glucose (FBG, 45.5%), family history of T2D (43.2%), increased body mass index (BMI, 36.4%), and high blood pressure (15.9%). A high proportion (70%) of participants with detected impaired FBG perceived they were at low risk of developing T2D. Participants with a family history of T2D (mean rank = 24.2) perceived the seriousness of T2D at a similar level as those without family history (mean rank = 21.2), with no significant difference (U = 205, *P *= 0.430). Nearly 30% of students did not feel confident they could prevent the development of T2D. Pearson’s correlations revealed direct relationships between perceived risk of T2D and BMI (*r* = 0.49, *P *= 0.001), fat mass percent (*r* = 0.51, *P *< 0.001), and waist circumference (*r* = 0.42, *P *= 0.005), and an inverse relationship was found with HDL-c (*r* = − 0.41, *P *= 0.005). The association of perceived risk of T2D with a family history of T2D revealed a trend toward significance (Chi-squared = 5.746, *P *= 0.057), and the association of perceived risk of T2D with physical activity was not significant (Chi-squared = 1.520, *P *= 0.468). The nutrition knowledge score was 74.32 ± 15.97 (recommended is > 75). However, knowledge scores regarding recommended intake of fruits, vegetables, high sodium foods, and whole grains to prevent T2D were only 36.36%, 34.09%, 47.73%, and 63.6%, respectively.

**Conclusions:**

The discordance between college students’ perceived risk and prevalence of T2D risk factors warrants strategies to address misperceptions of T2D risk and improve lifestyle behaviors among this study sample.

## Background

Type 2 diabetes (T2D) accounts for approximately 90–95% of all diagnosed cases of diabetes [1]. Previously considered a disease of middle- and older-adulthood, T2D is now highly prevalent among adolescents and young adults [2]. A multicenter study conducted to estimate changes in the prevalence of T2D in United States youths showed an increase among 10–20 years old [3]. The available literature has demonstrated an increasing prevalence of T2D among persons in the 30 s age bracket [4, 5]. These age groups represent the majority of college students in the United States. In addition to the current high incidence of T2D cases in different age groups, it has been reported that 84.1 million individuals in the ≥ 18 years age-group have prediabetes [1, 2]. The increasing incidence of these conditions observed in the younger age group have been attributed to lifestyle behaviors including poor nutritional choices and insufficient physical activity and the nonmodifiable risk factor, which is a family history of T2D [6–8]. Unhealthy lifestyle behaviors associated with being overweight and/or obese may promote impaired glucose utilization, high blood pressure, and dyslipidemia, which are in turn, strongly linked to an individual’s risk for the future development of T2D [9].

The college-age years are associated with tremendous sociobehavioral health changes that can promote the emergence of one or more T2D risk factors. While they are limited, studies have found that during their first year of school, 70% of college students are inclined to gain weight [10]. Moreover, marked increases in the prevalence of obesity among college students is evident by the end of the senior undergraduate year [11]. A multi-college student sample revealed that 23.3% did not exercise, and only 8.5% reported a daily intake of five or more servings of fruits and vegetables [12]. These dietary patterns and physical activity levels do not meet the recommendations of health experts. They could present a substantial problem for some college students who may already be at risk for T2D [13]. For instance, 61% of a sample of 660 college students reported a family history of T2D [14]. However, many college students rarely request annual checkups and testing for blood glucose, cholesterol, or blood pressure [15]. Few studies have shown that abnormal glucose levels and lipid profiles are consistently prevalent among college students [16, 17].

Furthermore, many young adults are unaware of their condition or their risk for T2D due to misinformation and a lack of medical care, which intensifies the likelihood of development of complications [1, 18]. Public health prevention and intervention programs have shown that among high-risk individuals, increased awareness, early detection of T2D risk factors, and even moderate lifestyle modifications may help slow down or prevent the onset of disease [18]. Thus, a higher personal perception of risk may lead to the adoption of a healthier lifestyle. By contrast, lower perceptions of risk may create challenges in preventive health behavioral interventions. Accordingly, individuals’ acceptance of preventative health messages may be influenced by their perception of the risk of developing T2D [19]. It is imperative to identify college students’ risk perception and to conduct mass screening programs and awareness to identify T2D risk factors at an early stage [4, 18, 19]. One of the most widely used health-related behavior modification theories studying perception is the Health Belief Model [HBM] [20]. It is a guiding framework that is used in health behavior interventions. Three HBM concepts that have been applied with regards to perception responses are self-efficacy, perceived susceptibility, and perceived severity. Self-efficacy is defined as one’s confidence in performing a particular behavior at a certain competency level. Perceived susceptibility refers to one’s perception of the risk of contracting a condition. Perceived severity is one’s perception of how serious a disease and its consequences are.

Consequently, assessing perceptions and T2D risk factors among college students is crucial for the successful promotion of disease prevention and intervention applications. Despite the high-risk lifestyle behaviors among college students, to date, there have been few studies that have examined their T2D risk and perceptions of the disease. As the prevalence of T2D rises among young adults, it is a significant public health priority to better identify and understand metabolic dysfunction in high-risk populations. The purpose of this study was to assess T2D risk factors and perception of the disease among college students at an upstate New York college. To the best of our knowledge, no studies of this nature have been conducted in this region.

## Methods

### Study population and recruitment

For this pilot study, a cross-sectional study design was used to generate preliminary data on T2D risk factors among 44 students at an upstate New York midsize public college (student enrollment, approximately 6000 as of spring 2017), and we further examined students’ perception of the disease. The inclusion criteria that were established in the study were college students ≥ 18 years who were enrolled in face-to-face undergraduate programs on campus; having a contact telephone and email; and signature on an informed consent form and a willingness to participate in the study. The exclusion criteria were being pregnant or self-described diabetes. An advertisement with details of the study purpose, risks, and benefits was sent to the entire student body through a campus email bulletin and myportal system to recruit study participants. Flyers were also purposefully placed on notice boards and other vantage points throughout the campus. The researchers obtained ethical approval from the Institutional Review Board (IRB) of the State University of New York (SUNY) College at Oneonta before commencement of the study. All standard safety measures and processes for the ethical handling of human subjects were adhered to in this study. Signed informed consent was obtained from each participant before their participation in the survey or initiation of measurements.

### Data collection

The American Diabetes Association screening guidelines [2] were used to determine T2D risk factors.

- Survey questionnaires
  i. Perception constructs—researchers adopted a 13-item survey that was developed based on the Health Belief Model’s constructs (perceived susceptibility, perceived seriousness and self-efficacy/confidence) and literature review of questionnaires from related studies [6, 10, 11, 16, 17, 19] to examine students’ perception of T2D.
  ii. Sociodemographic and lifestyle information—participants’ age, gender, ethnicity, country of birth, place of residence, diabetes status, family history of diabetes, income, medication use, smoking status, alcohol use, vitamin use, marital status, years in college, and physical activity levels were self-reported on the questionnaire. To identify the risk factors, a self-reported family history of T2D was categorized as “Yes” or “No”. In contrast, physical activity was considered as low or inactive for participants who performed < 5 days/week of 30 min exercise.
  iii. Nutrition knowledge related to T2D—the knowledge questions focused on general nutrition knowledge, and specific questions focused on knowledge related to the recommended servings of fruits and vegetables and the association between diet and T2D. Corrected answers were assigned a score range of 0–100%. An adequate knowledge for corrected answers was considered at 75% or more [21].

Survey questionnaires were administered online through Survey Monkey with a provision for signed informed consent. Participants had an option at the end of completing the questionnaire to schedule a day and time for anthropometric and metabolic profile information to be collected in a private room on campus. On the day before their appointment, participants were reminded to engage in an overnight fast of at least 8 h, not engage in any vigorous exercise and be well-hydrated. The collected information included:

b. Anthropometric
  i. Height and weight—height was measured to the nearest 0.1 cm without shoes and head adjusted to the Frankfurt plane on a calibrated stadiometer (Seca, Hamburg, Germany). Body weight was measured with the Bod Pod machine (see description under body composition and body weight). Body mass index (BMI) was then calculated as weight (kg)/height (m^2^). A BMI of ≥ 25 kg/m^2^ was classified as overweight or obese.
  ii. Waist and hip circumferences and waist-to-hip ratio—waist circumference (WC, cm) was measured with a nonstretchable tape midway between the lower rib margin and the upper end of the iliac crest. A waist circumference over 88 cm in women or over 102 cm in men was considered abdominal obesity. Hip circumference (cm) was measured at the widest point of the hip and together with the waist circumference was used to calculate the waist-to-hip ratio; a ratio above 0.85 in women and above 0.90 in men was classified as a risk for development of T2D.
  iii. Body composition and body weight—the BOD POD^®^ machine (COSMED USA, Inc., California) was used for body composition assessments for fat mass (%) and determined body weight. The BOD POD is a piece of innovative equipment that is based on the whole body displacement of air (Air Displacement Plethysmograph) to determine body composition. Its accuracy in measurement is similar (within 1% agreement with body fat) with underwater weighing. Additionally, an average test–retest variation of ± 2% body fat for the BOD POD has been demonstrated [22]. The participants were informed at the end of the survey questionnaire, and on the informed consent form, of the various measurements, what the visit to the research office would include and the attire requirements for the BOD POD measurements. To dress appropriately comprised wearing only compression shorts for men or a swimsuit for women. Before entry into the machine, jewelry was removed, and a swim cap was worn during testing. Each participant was measured twice and the results averaged. The BOD POD was calibrated each morning prior to use.
c. Metabolic profile

Fasting plasma glucose (FBG) levels and lipids [low-density lipoprotein cholesterol (LDL-c), high-density lipoprotein cholesterol (HDL-c), total cholesterol (TC), and triglycerides (TG)] were screened with the CardioChek^®^ Plus Analyzer device (PTS Diagnostics USA, Indiana). This point-of-care device’s accuracy in determining glucose and lipid levels is comparable to existing technology and has CLIA-waived status [23]. A fingerstick blood sample (40 µL) was applied to a strip with enzymatic and solid-phase methodology inserted into a reader, and the results were available in 2–5 min. FBG levels of ≥ 100 mg/dL, LDL-c of ≥ 100 mg/dL, HDL-c of < 60 mg/dL, TC of ≥ 200 mg/dL, and TG of ≥ 150 were considered impaired or abnormal. A blood pressure sphygmomanometer (Omron BP652N 7 Series, Omron Healthcare Inc. IL, U.S.) was used to screen participants for high blood pressure. A systolic blood pressure (SBP) and diastolic blood pressure (DBP) of > 130 mmHg and > 80 mmHg, respectively, were considered as high blood pressure.

Trained research assistants performed measurements using standardized calibrated instruments and procedures to ensure the reliability of the obtained data. In addition, two nutrition experts and one media communication expert reviewed the questionnaires before pre-testing among five students for validity and reliability. Participants with out of range values were advised to follow up with their physicians.

### Statistical analyses

Descriptive statistics was used to characterize the participants, and continuous variables were expressed as the mean ± standard deviation or frequencies and percentages for categorical variables. A Mann–Whitney U test was used to compare differences in the perceived seriousness of T2D between participants with and those without a family history of T2D. Pearson’s correlation was used to determine the relationships between the perception of T2D with risk factors, whereas a Chi square test was used for the categorical variables, physical activity, and family history. The statistical package SPSS version 25.0 (SPSS, IBM^®^ SPSS^®^ Statistics 25) was used for statistical analyses. A 0.05 significance level was used for all statistical tests, and *P*-values were two-sided.

## Results

Of a total of 132 non-diabetic college students who submitted an online survey questionnaire, 44 visited the research office to have their anthropometric and metabolic profile measured, and the data were included in the cross-sectional analysis to fit the purpose of the study.

- Sociodemographic and lifestyle characteristics

The mean age was 21.2 ± 7.2 years old, 79.5% were female, 88.6% were non-Hispanic white, 65.9% lived on campus, 97.7% were single, 65.9% were unemployed, 95.5% had a monthly income of less than $1000, and 34.1% were freshmen. Overall, 61.4% reported exercising less than 5 days a week for 30 min, making physical inactivity the most common T2D risk factor among the study sample. Additionally, 43.2% had a family history of T2D (Table 1).

**Table 1:** Sociodemographic and lifestyle characteristics of study participants

Variable	Mean ± SD	Frequency n (%)
Age	21.2 ± 7.2	21.2 ± 7.2
Gender (female)		35 (79.5)
Ethnicity (non-Hispanic White)		39 (88.6)
Year of college		
Freshman		15 (34.1)
Junior		9 (20.5)
		7 (15.9)
Sophomore		
Senior		13 (29.5)
Grade (GPA)	3.1 ± 0.8	
Income < $1000 (yes)		42 (95.5)
Marital status: single (yes)		43 (97.7)
Work status: unemployed (yes)		29 (65.9)
Residence: campus hall (yes)		29 (65.9)
Physical activity (30 + min < 5 days/week)		27 (61.4)
Family history of T2D (yes)		19 (43.2)

Data were expressed as mean ± standard deviation (SD) or frequency (%). *P*-value is significant at < 0.05

*GPA* grade point average, *T2D* type 2 diabetes

Participants had relatively adequate general nutrition knowledge, with a score of 74.32%. The respondents’ knowledge about recommended consumption of fruits and vegetables was low, at 36.36% and 34.09%, respectively. Only 47.73% were able to identify foods that were likely to be highest in sodium, while 63.6% of participants answered that T2D could be prevented by consuming more whole grains.

b. Anthropometric features

Table 2 shows that the BMI of the sample was 24.9 ± 6.1 kg/m^2^; with regards to T2D risk factor classification, 36.4% had increased BMI (overweight or obese). The waist circumference, waist-to-hip ratio, and fat mass percent of the study participants were determined as 81.1 ± 15.8 cm, 0.8 ± 0.1, and 24.9 ± 9.4%, respectively.

**Table 2:** Anthropometric features of study participants

Variable	Mean ± SD	Frequency n (%)
BMI (kg/m^2^)	24.9 ± 6.1	
BMI (≥ 25 kg/m^2^)		16 (36.4)
WC (cm)	81.1 ± 15.8	
Waist/hip ratio	0.8 ± 0.1	
Fat mass (%)	24.9 ± 9.4	

Data were expressed as mean ± standard deviation (SD) or frequency (%). *P*-value is significant at < 0.05

*BMI* body mass index, *WC* waist circumference

c. Metabolic profile measurements

**Fig. 1:**
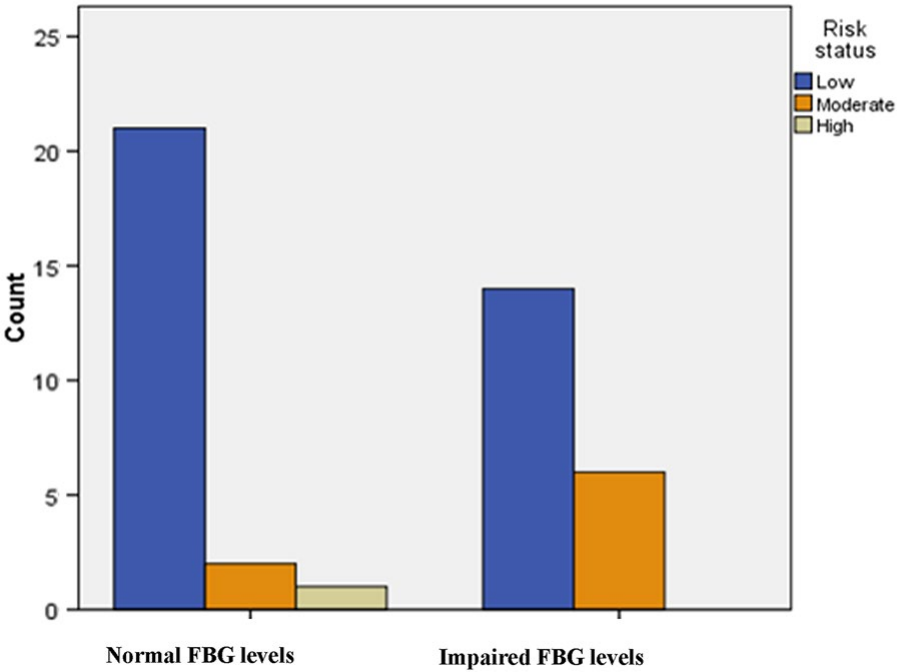
Perceived risk of developing type 2 diabetes by categories of fasting glucose status. *FBG* fasting blood glucose

Table 3 shows that of the study participants, 56.8% had low HDL-c levels, 45.5% had impaired fasting glucose levels, and 15.9% had high blood pressure. Figure 1 demonstrates that 70% of students (n = 20) who were detected to have impaired fasting glucose levels responded that they were at a low risk of developing T2D.

**Table 3:** Metabolic profile of study participants

Variable	Mean ± SD	Frequency n (%)
HDL-c (mg/dL)	57.9 ± 12.0	
HDL-c (< 60 mg/dL)		25 (56.8)
LDL-c (mg/dL)	82.6 ± 23.9	
TG (mg/dL)	98.7 ± 38.4	
TC (mg/dL)	160.3 ± 28.5	
FBG (mg/dL)	99.4 ± 8.5	
FBG (≥ 100 mg/dL)		20 (45.5)
SBP (mmHg)	116.3 ± 13.6	
DBP (mmHg)	71.6 ± 9.8	
High blood pressure (> 130/80 mmHg)		7 (15.9)

Data were expressed as mean ± standard deviation (SD) or frequency (%). *P*-value is significant at < 0.05

*HDL-c* high density lipoprotein cholesterol, *LDL-c* low density lipoprotein cholesterol, *TG* triglycerides, *TC* total cholesterol, *FBG* fasting blood glucose, *SBP* systolic blood pressure, *DBP* diastolic blood pressure

For the Pearson correlations, perceiving oneself at risk of T2D was positively correlated with BMI (r = 0.49, *P *= 0.001), fat mass percentage (r = 0.51, *P *< 0.001), and WC (r = 0.42, *P *= 0.005), whereas a significant inverse relationship was found with HDL-c (r = − 0.41, *P *= 0.005). Moreover, a Chi-square test of the association of perceived risk of T2D with a family history of T2D revealed a trend toward significance (Chi-squared = 5.746, *P *= 0.057), and an association of perceived risk of T2D with physical activity was not significant (Chi-squared = 1.520, *P *= 0.468) (Table 4).

**Table 4:** Associations of perceived risk with T2D risk factor measures of study participants

Variable	*r*	*Chi*-*squared*	*P*-value
HDL (mg/dL)	− 0.414		*0.005*
LDL (mg/dL)	0.023		0.882
TG (mg/dL)	0.228		0.136
TC (mg/dL)	− 0.099		0.523
FBG (mg/dL)	0.170		0.271
SBP (mmHg)	0.141		0.361
DSP (mmHg)	0.225		0.141
Fat mass (%)	0.506		*< 0.001*
BMI (kg/m^2^)	0.488		*0.001*
WC (cm)	0.416		*0.005*
Waist/hip ratio	0.257		0.093
Family history of T2D		5.746	0.057
Physical activity		1.520	0.468

Italic values indicate significance of *P*-value (*P* < 0.05)

*HDL-c* high density lipoprotein cholesterol, *LDL-c* low density lipoprotein cholesterol, *TG* triglycerides, *TC* total cholesterol, *FBG* fasting blood glucose, *SBP* systolic blood pressure, *DBP* diastolic blood pressure, *BMI* body mass index, *WC* waist circumference, *T2D* type 2 diabetes

Surprisingly, students with a family history of T2D (n = 19, mean rank = 24.2) perceived the seriousness of the disease at a similar level on average compared to those without a family history (mean rank = 21.2), but the difference was not statistically significant (U = 205, Z= − 0.789, *P *= 0.430). A proportion of 29.6% of students perceived that they did not have the self-efficacy or confidence to be able to prevent the development of T2D.

## Discussion

The most predominant T2D risk factor was physical inactivity (61.4%), which was significantly higher than the estimated 53.8% prevalence reported nationally among United States college students [12]. Similar findings of college students not meeting the recommended amount of physical activity were reported by other studies conducted in the United States and internationally [16, 19, 24]. Physical inactivity is an important modifiable risk factor that has been implicated in the development of T2D. In a meta-analysis, Smith et al. [8] reported that engaging in the minimum recommended amount of physical activity has potentially significant benefits to reduce the risk for T2D by 26%, compared with inactive individuals. In addition to physical inactivity, low HDL-c levels, impaired fasting glucose levels, a family history of T2D, and increased BMI (overweight or obese) were also prevalent among the six T2D risk factors we measured in our study, which is in line with studies on the subject [10, 17, 25]. The American Diabetes Association (ADA) recommends screening asymptomatic younger adults aged < 45 years with a BMI ≥ 25 kg/m^2^ who have at least one additional risk factor and for any individual ≥ 45 years irrespective of other risk factors [2]. Along with being overweight or obese, the risk factors in the ADA guidelines include having hemoglobin A1C > 5.7% or impaired glucose tolerance, acanthosis nigricans, cardiovascular disease, family history of T2D, low HDL-c, hypertension, physical inactivity, polycystic ovary syndrome, and history of gestational diabetes, being a member of ethnic minority group, or giving birth to a baby of > 9 lb. The population sampled for the study to institute the ADA T2D screening guidelines included those aged 18–44 years old [2]. Our findings align with the current ADA T2D recommendations that are based on the T2D risk factors identified in our study population and the age group represented in our sample that met the criteria for such screenings.

In addition, the participants’ general nutrition knowledge about their daily consumption of fruits and vegetables, foods high in sodium, and whole grain consumption to prevent T2D were deficient according to the Dietary Guidelines for Americans [12, 13]. A higher daily intake of fruits and vegetables [26, 27] and physical activity [28] have been well-recognized to be associated with a decrease in chronic disease burden and an increase in quality of life. The findings supported a similar trend to the reported data collected in other studies [6, 17, 29]. Excess weight is a complex interaction of initiating factors, including physical inactivity, unhealthy dietary patterns, and other environmental and genetic factors [7]. Thus, an active lifestyle may generate positive effects to improve overweight or obesity and simultaneously lower fasting glucose levels and enhance HDL-c levels [9, 30]. While the prevalence of overweight or obesity was lower compared to those revealed in other studies [10, 12, 17, 19, 25], it still warrants attention for the implementation of preventative strategies given the high rate of physical inactivity and nutrition knowledge deficit in our sample.

Family history is a well-established T2D risk factor, and this cause is largely unpreventable. According to Valdez et al. [31], people with a moderate to high family background of T2D and without other risk factors could have relative risks for developing T2D that are 2.3–5.5 times higher than individuals without a familial history. Since increased awareness, early health interventions, and lifestyle modifications are demonstrated approaches to lower the risks of T2D, making this population more aware of their family history of T2D may be a timely prevention tool. A study conducted by Ha and Caine-Bish [32] showed that the college setting serves as a great vehicle to promote healthy behavioral campaigns, and this could be used to motivate college students to engage in physical activity, follow a healthy dietary pattern, and maintain a healthy weight.

Our study shows that 70% of participants with impaired fasting glucose levels believed that they were at a low risk of developing T2D. Moreover, students with a family history of T2D perceived the seriousness of the disease at a similar level as those without a family history, but the difference for this was not statistically significant. The bias in underestimating one’s risk for chronic diseases is a known phenomenon since it depends on susceptibility to negative health effects and deficient previous experience with an illness [33]. For T2D, a potential explanation for the bias may be that those with a family history may have access to effective management strategies, including ease of monitoring the condition. In contrast, according to Weinstein [34], people with a family history of T2D are more likely to have a substantial perceived risk, and thus this bias is barely observed because of assumed sufficient previous experience and perceived uncontrollability among family members. While our findings of the students’ underestimated risks confirmed those of previous studies [14, 35–37], it is still disturbing and may qualify target groups for diabetes awareness education and interventions for risk factors. Individuals must acknowledge their susceptibility to disease risks, which is a sign of readiness for behavior change, to tailor prevention programs to meet such needs [38]. The conflicting results on perceived and T2D risk factors among college students illustrate the need for more research to be conducted in this area.

Based on the Health Belief Model, a high perceived risk of disease or perceived susceptibility and perceived severity are critical components in predicting whether a person can gain the self-confidence and implement healthy behaviors to reduce the risk of disease [20]. In our sample, nearly 30% of the participants were unsure or disagreed when asked if they were confident of preventing T2D. Again, this is disturbing given that 61 percent do not exercise, 36 percent are overweight or obese, and a considerable number are deficient in nutrition knowledge related to T2D, among the other risk factors that were identified in this sample. This reveals that students do not possess adequate education in the prevention of T2D because they seem to be uninformed that their present lifestyle may be placing them at risk of T2D.

The strengths of the current study are that first, a theoretical model (the Health Beliefs Model) informed it. Second, it utilized multiple forms of data (survey data and anthropometric measures) and included objective metabolic measures (lipid profiles, fasting plasma glucose, and blood pressure) to assess T2D risk. The small sample size of 44 students who were conveniently recruited from a mid-size college campus is a key limitation of this pilot study, and the findings cannot be generalized. Based on the IRB regulations, potential participants were made aware through the study questionnaire, informed consent, and reminders to visit the research office of what measurements would be taken and what they had to do, including the dress requirement for the BOD POD measurement. Although researchers contacted all who submitted the online questionnaire (n = 132) to visit to take their measurement, only 44 individuals reported. The availability of the data on the measures of T2D risk factors for those who did not attend would have strengthened our findings. In addition to the concerns already stated, the single center design of our study is a considerable weakness, and the extent of extrapolation is limited to the specific study population. These results should be considered cautiously because the study relied on self-reporting for questions on physical activity, family history of T2D, and 24-h recall for food intake and could be subject to recall bias.

## Conclusions

College students in this study sample were unaware of their risk, were overly positive about their own health, and considerably underrated their risk of developing T2D or seriousness of the disease. Moreover, 30% did not feel confident that they could prevent T2D. These findings point to a greater need to effectively address the students’ misconceptions and increase the awareness of T2D and its risk factors in this particular sample to promote early detection and healthy lifestyle behavior changes.

## Data Availability

The datasets analysed for this study are available from the corresponding author upon reasonable request.

## Abbreviations

T2D: Type 2 diabetes
HDL-c: High density lipoprotein cholesterol
LDL-c: Low density lipoprotein cholesterol
TC: Total cholesterol
TG: Triglycerides
FBG: Fasting blood glucose
BMI: Body mass index
WC: Waist circumference
SBP: Systolic blood pressure
DBP: Diastolic blood pressure
HBM: Health Belief Model
SUNY: State University of New York
IRB: Institutional Review Board
